# Access to hip and knee arthroplasty in England: commissioners’ policies for body mass index and smoking status and implications for integrated care systems

**DOI:** 10.1186/s12913-022-08999-9

**Published:** 2023-01-24

**Authors:** Joanna McLaughlin, Joshua Elsey, Ruth Kipping, Amanda Owen-Smith, Andrew Judge, Hugh McLeod

**Affiliations:** 1 Musculoskeletal Research Unit, Translational Health Sciences, Bristol Medical School, University of Bristol, Learning and Research Building, Level 1, Southmead Hospital, Bristol, BS10 5NB UK; 2 Bristol Medical School, University of Bristol, 5 Tyndall Avenue, BS8 1UD Bristol, UK; 3 Population Health Sciences, Bristol Medical School, University of Bristol, Bristol, BS8 2PS UK; 4 National Institute for Health Research Bristol Biomedical Research Centre, University Hospitals Bristol and Weston NHS Foundation Trust and University of Bristol, Bristol, UK; 5 Nuffield Department of Orthopaedics, Rheumatology and Musculoskeletal Sciences (NDORMS), University of Oxford, Oxford, UK; 6 National Institute for Health Research Applied Research Collaboration West (NIHR ARC West), University Hospitals Bristol NHS Foundation Trust, Bristol, BS1 2NT UK

**Keywords:** Body mass index, Smoking, Arthroplasty, Health optimisation, Healthcare policy

## Abstract

**Background:**

Commissioning policies are in place in England that alter access to hip and knee arthroplasty based on patients’ body mass index and smoking status. Our objectives were to ascertain the prevalence, trend and nature of these policies, and consider the implications for new integrated care systems (ICSs).

**Methods:**

Policy data were obtained from an internet search for all current and historic clinical commissioning group (CCG) hip and knee arthroplasty policies and use of Freedom of Information (FOI) requests to each CCG. Descriptive analyses of policy type, explicit threshold criteria and geography are reported. Estimates were made of the uptake of policies by ICSs based on the modal policy type of their constituent CCGs.

**Results:**

There were 106 current and 143 historic CCGs in England at the time of the search in June 2021. Policy information was available online for 56.2% (140/249) CCGs. With the addition of information from FOIs, complete policy information was available for 94.4% (235/249) of CCGs. Prevalence and severity of policies have increased over time. For current CCGs, 67.9% (72/106) had a policy for body mass index (BMI) and 75.5% (80/106) had a policy for smoking status for hip or knee arthroplasty. Where BMI policies were in place, 61.1% (44/72) introduced extra waiting time before surgery or restricted access to surgery based on BMI thresholds (modal threshold: BMI of 40 kg/m^2^, range 30–45). In contrast, where smoking status policies were in place, most offered patients advice or optional smoking cessation support and only 15% (12/80) introduced extra waiting time or mandatory cessation before surgery. It is estimated that 40% of ICSs may adopt a BMI policy restrictive to access to arthroplasty.

**Conclusions:**

Access policies to arthroplasty based on BMI and smoking status are widespread in England, have increased in prevalence since 2013, and persist within new ICSs. The high variation in policy stringency on BMI between regions is likely to cause inequality in access to arthroplasty and to specialist support for affected patients. Further work should determine the impact of different types of policy on access to surgery and health inequalities.

**Supplementary Information:**

The online version contains supplementary material available at 10.1186/s12913-022-08999-9.

## Background

Hip and knee osteoarthritis is a leading contributor to the global burden of disease [1, 2] and hip and knee arthroplasty are two of the most common high-cost elective surgical procedures provided in the National Health Service (NHS) [3]. The initial management pathway of osteoarthritis [4] involves advice on therapeutic exercise and weight management, followed by topical, oral and transdermal analgesia. If self-management and analgesia are not effective, the third line of treatment is referral to specialist care where patients may be considered for arthroplasty. In England, National Institute for Health and Care Excellence (NICE) guidance (2008,2014 and 2022) for people with osteoarthritis promotes self-management to achieve positive behavioural changes including the offer of interventions to support weight loss for patients who are overweight or obese [4–6]. In 2015, NICE’s osteoarthritis quality standard specified that commissioners “ensure that they commission services in which adults with osteoarthritis are not referred for consideration of joint surgery until they have been supported with non‑surgical core treatments for at least 3 months” [7].

Between 2013 and 2022, clinical commissioning groups (CCGs) were statutory NHS bodies with budgetary responsibility for the planning and commissioning of most health care services for their local population. CCGs set their own referral criteria for hip and knee arthroplasty leading to variation in policies across England, and these may include criteria for BMI (body mass index) and smoking thresholds. In some cases, ‘health optimisation’ is cited as the reason for policy introduction – whereby patients are offered extra support and/or an extra period of time before surgical referral to address weight management and smoking cessation in order to improve their overall health as well as their surgical outcome [8]. In other cases, policies are used to restrict access to surgery without necessarily offering health improvement opportunities, heightening the risk of increasing health inequalities through rationing of surgery and raising ethical concerns [9, 10]. Criteria for BMI and smoking governing access to hip or knee arthroplasty in end stage osteoarthritis affect many thousands of patients given that around 10% of adults will require a hip or knee arthroplasty in their lifetime [11], 64% of adults in England are obese or overweight [12] and 14% are current smokers [13]. The higher burden of obesity and smoking in more socioeconomically disadvantaged groups in society [12] means policies restricting access to surgery based on BMI and smoking status disproportionately impact people already experiencing health inequalities [14].

The Royal College of Surgeons reported that the proportion of CCGs with mandatory BMI upper thresholds for referral for hip and knee arthroplasty was 13% in 2014, rising to 22% in 2016 [9]. Further survey of CCGs by the Association of British HealthTech Industries [15] revealed that by 2017 this figure had risen again to 47%. The high prevalence of these policies is in spite of the position of the Royal College of Surgeons that all commissioning policies should be based on clinical need and “patient-specific factors (including age, sex, smoking, obesity and comorbidities) should not be barriers to referral for joint surgery” [16]. A decision published by an NHS Clinical Senate was that “NHS bans delaying surgery until patients stop smoking or lose weight are not supported and risk widening health inequalities” [17].

The 106 CCGs in England were replaced by 42 integrated care systems (ICSs) in 2022. This evolution marks a move away from local general practitioner membership bodies being responsible for strategic commissioning to one based on collaboration between organisations [18]. This change provides an opportunity to reassess the role of local commissioning policies to restrict access to hip and knee arthroplasty which have been inherited from CCGs by the ICSs. We collated policy information from each current and historic CCG and assess the extent to which policies inherited by the new ICSs will place limitations on access to arthroplasty based on BMI and smoking.

## Methods

### Aims

The aims of this study were to ascertain the prevalence, trend and nature of commissioning policies in use in England that alter access to hip and knee arthroplasty surgery based on patients’ body mass index or smoking status, and consider the implications for new integrated care systems (ICSs).

### CCG policy documents

Lists of CCGs in existence by calendar year were obtained from information published online by the Office for National Statistics [19]. We searched for all published policies that related to NHS patient referral for elective hip and knee arthroplasty surgical opinion (including generic policies that address all referrals for elective surgery) in effect any time from January 2013 to June 2021. Each policy was specifically reviewed for BMI and smoking criteria. Other access criteria e.g., symptoms scoring, were not collated.

We defined ‘BMI policies’ and ‘Smoking policies’ as those policies with criteria designed to alter, limit or delay access to surgery for patients based on their identification as overweight or obese including via body mass index, or on their smoking status respectively. For each policy identified, the following data were recorded: start and end date, BMI or smoking status threshold, extra waiting time or other requirement for access to surgical referral, nature of support services offered to patients for weight management or smoking cessation, mandatory or optional elements of patient engagement with policy thresholds.

To identify the policies, a search protocol was developed and applied to each of the 106 current and 143 historic (formed at any point from 2013 but no longer in existence due to mergers) CCGs in England [19]. We searched CCG websites (formal websites for the organisations, of the format www.[CCG name].nhs.uk) and used search terms in an internet search engine where CCG websites were unavailable or held no reference to relevant policies. Where a first researcher was unable to locate any policy information for a CCG, a second researcher repeated the search. The searches were completed in May to June 2021.

### Freedom of information requests

To determine the policy situation for CCGs with no available online policy information, and to check the accuracy of the identified policy information, we sent Freedom of Information (FOI) requests to each current CCG by email in June 2021. The requests asked CCGs to verify or amend the data collected through our online searches. Repeat requests were sent to CCGs in July 2021 where they did not initially respond with information about their component historic CCGs.

### Analysis

Policies were categorised based on their criteria and content, ranging from advice to patients to denial of access to surgery. These six categories are further detailed in Table 1.

**Table 1:** Prevalence and content of hip and knee arthroplasty policies for body mass index and smoking (106 clinical commissioning groups in England 2021)

Policy type	Body mass index		Smoking	
	n	%	n	%
Policy situation unknown	0	0.0	1	0.9
Policy introduced but now inactive	11	10.4	4	3.8
No policy	23	21.7	21	19.8
Policy introduced and still active of which:	72	67.9	80	75.5
1 advice given only—no added service or extra wait	10	13.9	18	22.5
2 extra service provision—advised to use but no requirements	12	16.7	31	38.8
3 extra service provision—mandatory referral but no requirement to engage	6	8.3	19	23.8
4 extra waiting time to surgery, but no penalty for engagement or outcome	6	8.3	1	1.3
5 extra service provision—access to surgery delayed until engagement/outcome attempted	18	25.0	7	8.8
6 extra service provision—access to surgery denied until BMI threshold or smoking status is met	20	27.8	4	5.0

We report descriptive statistics on the prevalence and nature of policies examining BMI and smoking policies separately, as well as differentiating between historic and current policies.

For each ICS we identified the policy type likely to have been inherited from its constituent CCGs by determining the most common (modal) policy of the CCGs.

Mapping software (ArcMap Version 10.7.1) was used to present the geographical distribution of policies in CCGs in June 2021 and the estimated distribution of policies for ICS organisations in 2022 in choropleth maps. CCG regions with obesity and smoking prevalence higher than the England national average (as reported in the Public Health Outcomes Framework in 2021) [13] were indicated on the maps to allow assessment of association with policy severity.

## Results

### Data sources and data completeness

Policy information was available online for 84.9% (90/106) of current CCGs in June 2021. Many historic CCGs that subsequently merged into new CCGs did not have publicly available archived websites and so policy information was only available online for 35.0% (50/143) of historic CCGs. Our internet searches therefore returned policy information for 56.2% (140/249) CCGs overall.

Responses to FOI requests were received from 94.3% (100/106) of current CCGs. With prompting to include information about their constituent, historic CCGs, FOI information was gained for 92.0% (229/249) CCGs overall. One current CCG only provided partial information about their policies and one other current CCG had no policy information available online and did not respond to the FOI request. Policy information was unavailable or only partially available for 12 of the historic CCGs. In combination, the internet searches and FOI data gave complete policy information for 99.1% (105/106) of current CCGs and 94.4% (235/249) of all current and historic CCGs.

### Changes in policy prevalence and severity over time

**Fig. 1:**
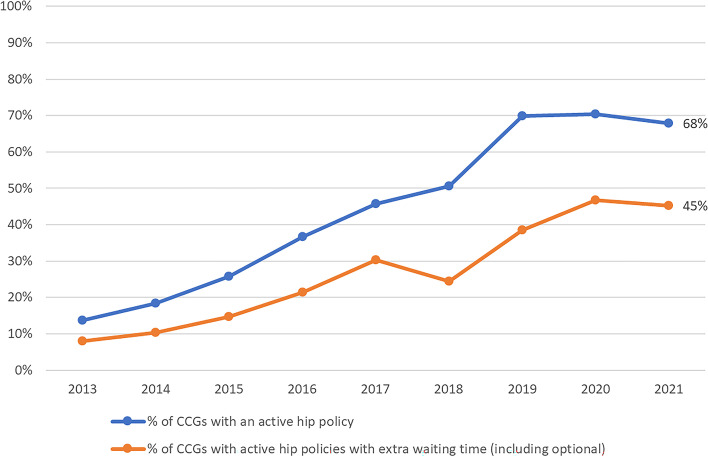
Percentage of clinical commissioning groups with a body mass index policy in place for hip arthroplasty by year (total *n* = 106 in 2021)

**Fig. 2:**
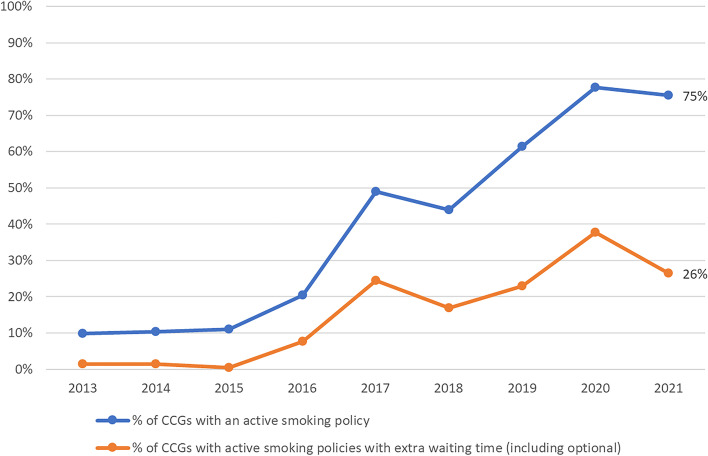
Percentage of clinical commissioning groups with a smoking status policy in place for hip arthroplasty by year (total *n* = 106 in 2021)

Between 2013 and 2021, the prevalence of BMI policies increased from 14 to 68% of CCGs (Fig. 1), and from 10 to 75% of CCGs for smoking (Fig. 2). Many of the mergers in CCGs occurred in 2018 and this resulted in a sharper increase in policy prevalence, as CCGs without policies tended to become subject to the policies of other constituent CCGs in the newly formed CCG. The stringency of these policies has generally increased over time. By 2021, 45% and 26% of CCGs were using policies that require extra waiting time before surgery for BMI and smoking respectively (Figs. 1 and 2).

### Prevalence and nature of policies in 2021

In June 2021, 84% (89/106) of CCGs had at least one active policy: 59% (63/106) had both a BMI and a smoking policy, 16% (17/106) had only a smoking policy and 9% (9/106) had only a BMI policy.

Policies ranged in severity from stating patients should be given advice on weight management or smoking cessation without any restriction in access to surgery, to denying access to surgery until patients meet a particular BMI threshold or provide evidence that they have quit smoking (Table 1).

#### Body mass index policies

Of the 72 policies in use in 2021 which alter, limit or delay access to surgery for patients based on their identification as overweight or obese, 86% (62/72) specified a BMI threshold, ranging from 25 to 45 kg/m^2^, in some cases only to identify which patients would be offered advice on weight management (Table 2). ‘Not specified’ indicates that all patients were subject to the same restrictions regardless of BMI. Common examples of this included access to lifestyle interventions for 3 months prior to surgery, thereby all patients would have the opportunity to improve an aspect of their lifestyle, including weight loss if appropriate. Thirty-seven percent (27/72) of these policies specified a BMI threshold for whether it was mandatory for a patient to engage with weight management or wait for extra time before surgery (Table 2). Notably, a third of the CCG policies require that patients engage with weight management or successfully lose weight to meet a certain threshold use thresholds ≤ 30 kg/m^2^. This means that overweight patients are impacted by the restrictions as well as obese patients.

**Table 2:** Use of body mass index (BMI) thresholds in weight management policies for hip and knee arthroplasty *n* = 72

Body mass index threshold	BMI threshold for policy application/ patient eligibility of any kind		BMI threshold for mandatory engagement or wait for access to surgery^a^		BMI threshold for access to surgery	
	*n* = 72		*n* = 27		*n* = 12	
	n	%	n	%	n	%
No specific threshold^b^	10	13.9	1	3.7	0	
25	15	20.8	9	33.3	0	0.0
30	10	13.9	1	3.7	1	8.3
35	27	37.5	6	22.2	2	16.7
40	7	9.7	7	25.9	6	50.0
45	3	4.2	3	11.1	3	25.0

^a^Includes mandatory participation with weight management services and/or mandatory weight loss to reach a % weight loss or a specified BMI threshold

^b^all patients were subject to the same restrictions regardless of BMI e.g. all patients given access to lifestyle improvement services for 3 months before surgery

Of the 72 policies in use in 2021, 61% (44/72) specified an extra waiting time requirement for accessing surgery. This extra waiting time was added to the patient pathway before they could be listed for surgery. When recorded, the range in extra waiting time was three months to one year, with six months the most common duration. In a quarter of these CCGs (12/44) patients must meet a particular BMI threshold before being able to access surgery rather than wait a specified extra time period.

#### Smoking policies

Seventy-five percent (80/106) of CCGs had a smoking policy for hip or knee arthroplasty in 2021, and 39% (31/80) of these policies required that patients accept referral for smoking cessation support, wait extra time before surgery or that patients must quit smoking to access surgery. Almost half these policies required that patients accept a referral for smoking cessation and/or engage with the service on offer but did not specify the duration of this engagement. For those policies that did specify the extra wait times to which patients would be subject, the range was 8 weeks to 6 months.

#### Geographical distribution of policy use

**Fig. 3:**
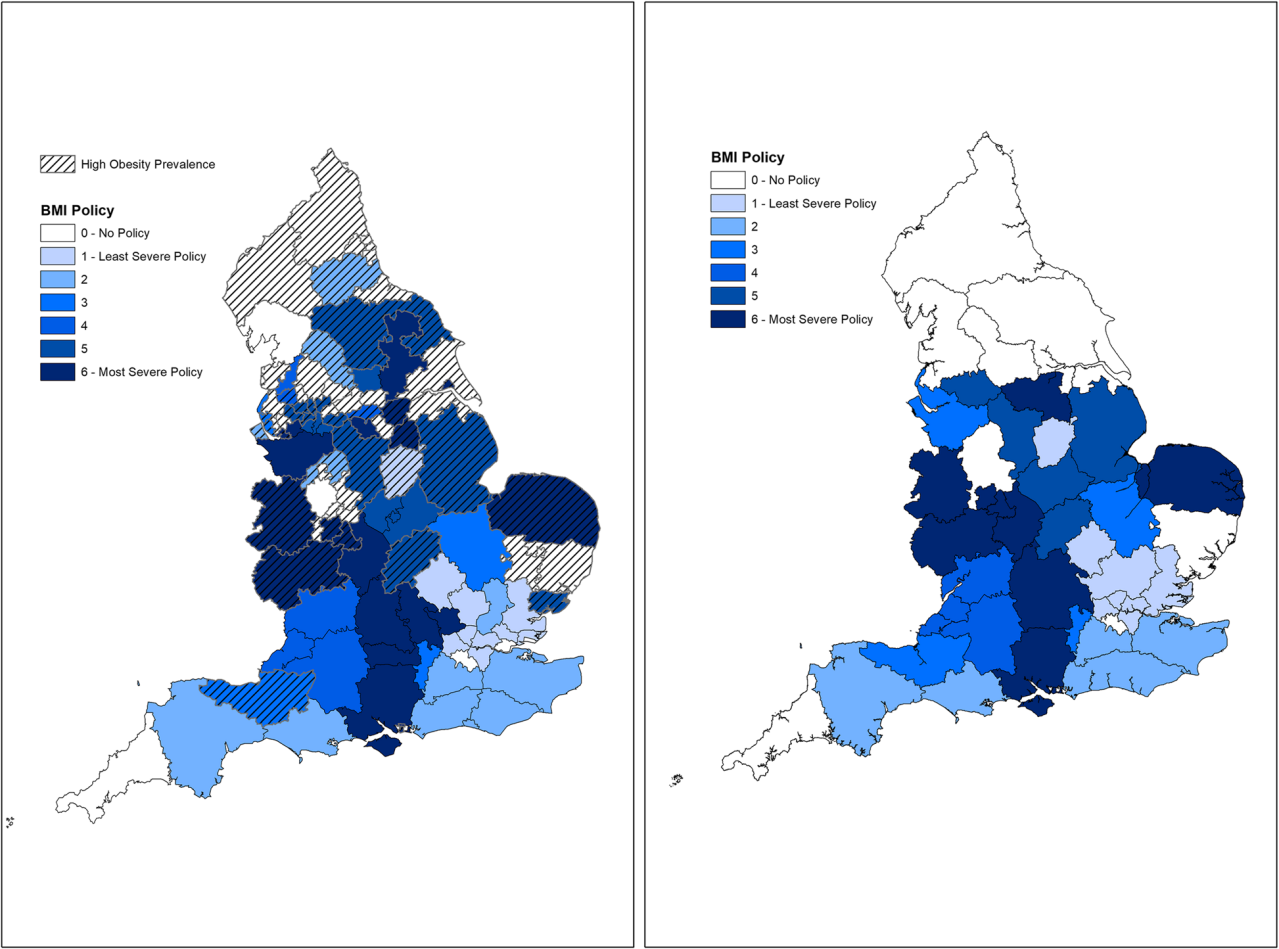
Left: clinical commissioning groups’ BMI policy prevalence and severity in 2021 overlain with population obesity prevalence*. Right: estimated integrated care system BMI policy prevalence and severity. * ‘high prevalence’ = higher than the England national average as reported in the Public Health Outcomes Framework in 2021 [13]

**Fig. 4:**
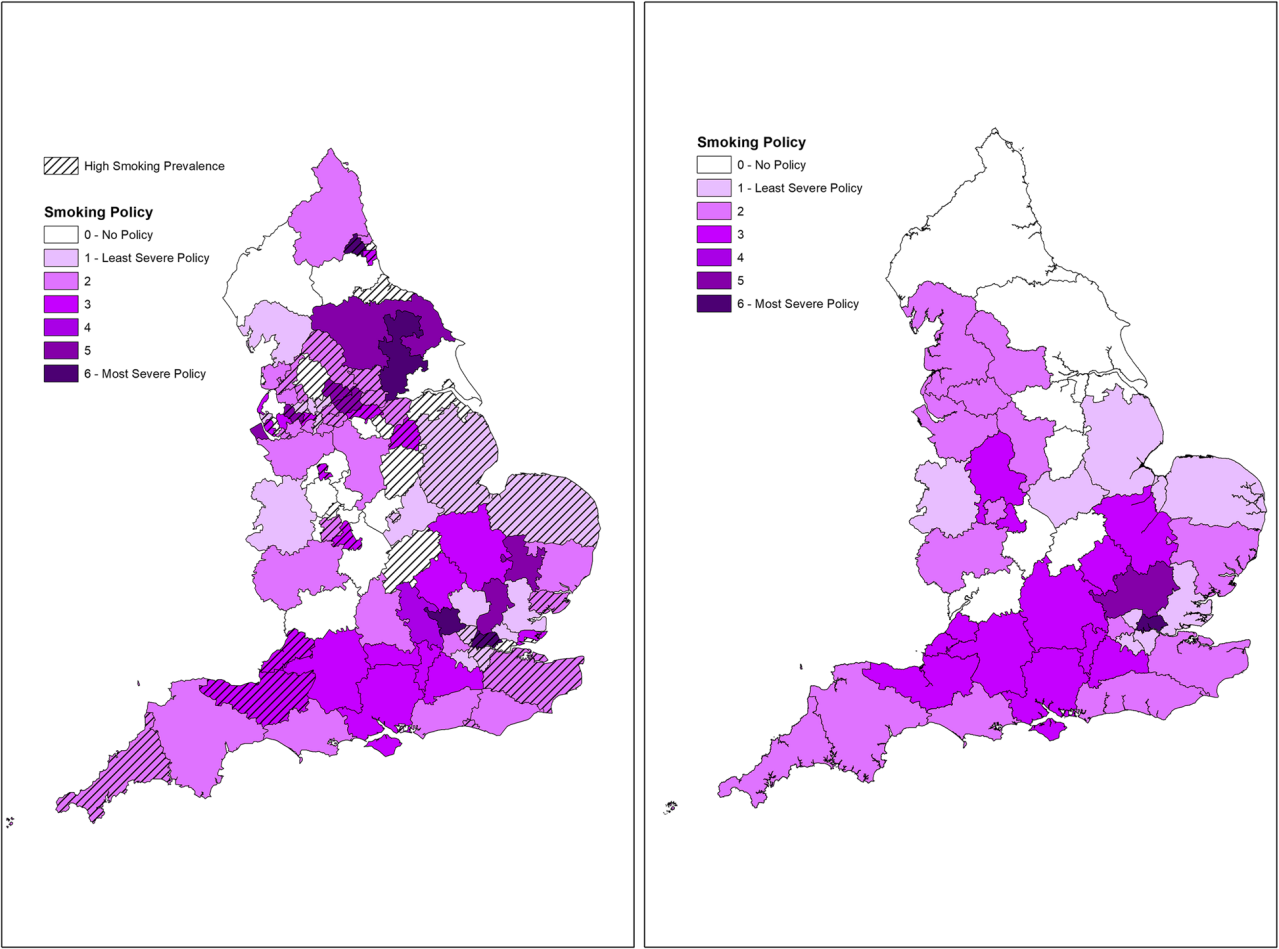
Left: clinical commissioning groups’ current smoking policy prevalence and severity in 2021 overlain with population smoking prevalence*. Right: estimated integrated care system smoking policy prevalence and severity. * ‘high prevalence’ = higher than the England national average as reported in the Public Health Outcomes Framework in 2021 [13]

There was marked geographical variation in the current BMI and smoking policies (Figs. 3 and 4 respectively). There was no association evident between the choice of policy severity and the population prevalence of obesity or smoking in CCGs. The figures also display the estimated uptake of these policies into the new ICS geography. For BMI policies: 40.5% (17/42) of ICSs will have a policy in place that mandates extra waiting time ahead of surgery or a requirement to meet a BMI threshold in order to access surgery (3 with category 4, 5 with category 5 and 9 with category 6). For smoking policies: 5% (2/42) of ICSs will have policies in place that do place restrictions on access to surgery (categories 4 to 6 inclusive).

## Discussion

### Summary of findings

Data on the prevalence and content of commissioners’ policies for BMI and smoking that determine access to elective hip and knee arthroplasty were available for 105/106 (99.1%) of current CCGs in 2021. Prevalence of policy use is high and rising: 68% and 75% of CCGs for BMI and smoking respectively in 2021. There is high variation in policy content and severity; with 42% and 11% of CCGs using policies that require extra waiting time or achievement of weight loss or smoking cessation before surgery for BMI and smoking respectively. Policy severity does not appear to be associated with high prevalence of obesity or smoking. We estimate that 40% of integrated care systems will have a policy in place that mandates extra waiting time ahead of surgery or a requirement to meet a BMI threshold in order to access surgery, and 5% will have such a restrictive policy for smoking.

Recent NICE guidance for osteoarthritis is explicit that people should not be excluded from referral for arthroplasty because of “smoking […] overweight or obesity” [4]. In this context, our findings that commissioners’ policies do not align with NICE guidance show that the restrictions in access to surgery imposed by many localities are problematic.

### Strengths and limitations

This study used a comprehensive search of all CCGs that achieved high data completeness and provided detailed information on the content and variation in policies. A limitation is that we can only estimate future ICS policy position as no formal mechanism for deciding which of the constituent CCG policies the ICS will retain is evident. With high variation in policy content, it is challenging to directly equate policy categories to conformity with relevant NICE guidelines.

### Relation to other work and further context

Variation in musculoskeletal commissioning policies [20] and arthroplasty access criteria [15, 21] have been previously documented in England, and BMI thresholds for arthroplasty are also in use internationally [22, 23]. The proportion of hip and knee arthroplasty patients with obesity or who smoke is 49%, and 11% respectively in the UK [24, 25], meaning policies with BMI criteria have implications for the majority of patients.

Access to elective surgery may be purposefully limited for patients who smoke or for patients with obesity through policies produced by healthcare commissioners. The available evidence indicates that hip and knee arthroplasty is cost-effective for almost every patient who receives treatment [26] and a recent analysis of the National Joint Registry found no evidence of poorer outcomes in arthroplasty patients with high BMI [27]. Sustained, significant weight loss is difficult to achieve and literature reports that very few patients denied access to arthroplasty due to their BMI go on to lose sufficient weight to qualify for surgery [28, 29]. BMI policies that limit access to surgery are therefore not justified on clinical grounds and instead risk widening health inequalities given that patients’ ability to pursue independently-funded treatment varies with their affluence [30]. Use of policies that require patients to lose weight before accessing surgery should be based in evidence, yet there is currently no strong evidence that pre-surgical weight loss improves surgical outcomes. Indeed, short-term pre-surgical weight loss raises concerns of deconditioning and post-surgical rebounds in weight in arthroplasty patients [31].

In contrast, evidence is mounting for the beneficial role of ‘prehabilitation’ for patients in the peri-operative period to improve their overall health [8, 32, 33]. These interventions include support for exercise and weight management but do not restrict access to surgery based on patient engagement or success.

Whilst it is healthcare commissioners who set surgery access policies, it is generally local authorities who commission community-based weight management and smoking cessation services. Financial pressures on local authorities in recent years have led to the reduction or decommissioning of these services in some regions [34]. This leaves a potential mismatch for patients facing a BMI or smoking threshold to access surgery and the access to services to support them in reaching these thresholds. Longstanding variation in both the surgery access policies and also in the support provided for weight management or smoking cessation in different regions is highly likely to be the cause of health inequalities. National mapping of weight management services in 2015 revealed that only 61% of local authorities had a ‘tier 2’ weight management service available. Additional funding for the financial year 2021/22 has supported local authorities in increasing their provision of weight management services, although the continuation in provision of these services without recurrent additional funding is not yet clear [35, 36]. The intended integration of commissioning and service delivery across health, care and community organisations resulting from the introduction of ICSs [37] may reduce the disconnect between healthcare commissioning policies targeting BMI and the regional alignment of resources for health improvement interventions.

## Conclusions

Integrated care systems have now replaced CCGs as the clinically-led statutory NHS bodies responsible for the planning and commissioning of health care services for their local area [37]. It is unclear as yet on their approach to setting policy where their constituent CCGs had differences in policies, but our estimates indicate that restrictive BMI policies governing access to hip and knee arthroplasty that are unsupported by NICE guidance will be in use in 40% of ICSs. It is our recommendation that ICS decision makers take this transition opportunity to ensure that integrated, complementary weight management and smoking cessation support services are available and to cease the use of restrictive BMI and smoking threshold policies for surgery. Further work is required to determine the scale of the impact these policies have on health inequalities and the optimum way in which to include health improvement in surgical pathways.

## Supplementary Information

**Additional file 1.**

**Additional file 2.**

## Data Availability

The accompanying supplementary material includes the search protocol. The data generated during this study are available from the corresponding author on reasonable request.

## Abbreviations

CCG: Clinical Commissioning Group
ICS: Integrated care system
FOI: Freedom of Information
BMI: Body mass index
