# Supplementary material for: Access to hip and knee arthroplasty in England: commissioners’ policies for body mass index and smoking status and implications for integrated care systems
Source: BMC Health Serv Res. 2023 Jan 24;23:77. doi: 10.1186/s12913-022-08999-9 (PMC9875525; doi:10.1186/s12913-022-08999-9)
Supplement: Supplementary file 1 — Additional file 1. [file 12913_2022_8999_MOESM1_ESM.docx]

# Supplementary Material: search protocol

## Objective:

1. Locate a copy of all relevant English clinical commissioning group (CCG) policies

## Inclusion criteria:

Policies that relate to National Health Service patient referral for elective hip and knee surgical opinion (this may include generic policies that address all referrals for elective surgery within the CCG) in effect any time from 1^st^ January 2009 to date.

**Policy search terms:** Hip and knee surgery, elective surgery, joint replacement surgery, arthroplasty, elective orthopaedic surgery, pre surgical health optimisation, prehabilitation, presurgical weight loss or smoking cessation, BMI/body mass index and or smoking criteria for surgery, BMI/weight/overweight/obesity/smoking eligibility or thresholds for surgical referral

## Exclusion criteria:

Policies for specific operations other than arthroplasty, or conditions only, such as osteoporosis

## Search method:

1. For each CCG:
   1. Locate the CCG website (may be archived if no longer in operation)
   2. Search for the relevant policy(ies) on the website to cover the whole period the CCG was in operation
   3. If unable to locate the policies via the CCG website, use an internet search with the search terms listed above to locate the policy document directly
2. Where a first researcher is unable to locate the policy, a second researcher repeats the search
3. Proceed to Freedom of Information Request to verify policy information where this has been retrieved and to request missing policy information.
