# Supplementary material for: Access to hip and knee arthroplasty in England: commissioners’ policies for body mass index and smoking status and implications for integrated care systems
Source: BMC Health Serv Res. 2023 Jan 24;23:77. doi: 10.1186/s12913-022-08999-9 (PMC9875525; doi:10.1186/s12913-022-08999-9)
Supplement: Supplementary file 2 — Additional file 2. [file 12913_2022_8999_MOESM2_ESM.docx]

Supplementary Material

We searched 10 CCG websites to determine the key search terms that returned relevant policy information.

Policy search terms:

Hip and knee surgery, elective surgery, joint replacement surgery, arthroplasty, elective orthopaedic surgery, pre surgical health optimisation, prehabilitation, presurgical weight loss or smoking cessation, BMI/body mass index and or smoking criteria for surgery, BMI/weight/overweight/obesity/smoking eligibility or thresholds for surgical referral.”
